# Animal Models in Diabetic Research—History, Presence, and Future Perspectives

**DOI:** 10.3390/biomedicines11102852

**Published:** 2023-10-20

**Authors:** Shashank Pandey, Tomas Chmelir, Magdalena Chottova Dvorakova

**Affiliations:** 1 Biomedical Center, Faculty of Medicine in Pilsen, Charles University, 323 00 Pilsen, Czech Republic; shashank.pandey@lfp.cuni.cz; 2 Department of Pharmacology and Toxicology, Faculty of Medicine in Pilsen, Charles University, 323 00 Pilsen, Czech Republic; 3 Department of Physiology, Faculty of Medicine in Pilsen, Charles University, 323 00 Pilsen, Czech Republic; tomas.chmelir@lfp.cuni.cz

**Keywords:** diabetes mellitus, animal model, history, organ-on-chip

## Abstract

Diabetes mellitus (DM) is a very serious disease, the incidence of which has been increasing worldwide. The beginning of diabetic research can be traced back to the 17th century. Since then, animals have been experimented on for diabetic research. However, the greatest development of diabetes research occurred in the second half of the last century, along with the development of laboratory techniques. Information obtained by monitoring patients and animal models led to the finding that there are several types of DM that differ significantly from each other in the causes of the onset and course of the disease. Through different types of animal models, researchers have studied the pathophysiology of all types of diabetic conditions and discovered suitable methods for therapy. Interestingly, despite the unquestionable success in understanding DM through animal models, we did not fully succeed in transferring the data obtained from animal models to human clinical research. On the contrary, we have observed that the chances of drug failure in human clinical trials are very high. In this review, we will summarize the history and presence of animal models in the research of DM over the last hundred years. Furthermore, we have summarized the new methodological approaches, such as “organ-on-chip,” that have the potential to screen the newly discovered drugs for human clinical trials and advance the level of knowledge about diabetes, as well as its therapy, towards a personalized approach.

## 1. Introduction

Since time immemorial, scientists have been trying to reveal the secrets of how living organisms function. They have discovered the causes and mechanisms of the diseases through animal models/tests. Experiments on animals have played a critical, fundamental, and irreplaceable role in the research of the functioning of individual tissues, organs, and organ systems. Animal models of various human diseases have been created, which has greatly helped to understand the pathophysiological processes associated with the particular disease, and these models are still widely used in research. The importance of animal models in research is also indicated by the fact that most of the important research centers have their own full-fledged animal facilities for the development and breeding of these models [1].

The analogy between humans and laboratory animals lies in their physiological processes, which may allow researchers to use a disease model for research purposes. A suitable animal model can be found throughout the animal kingdom. Practice shows that even a species that is genetically and taxonomically very distant from humans, such as the fruit fly, can be a good laboratory model for understanding the basic principles of signaling or cellular and developmental biology. The term “animal model” is sometimes misunderstood because humans are not modeled. The degree of similarity of a particular model to a person is referred to as fidelity. A high-fidelity model is very close to a human and can provide valuable information, but it is very difficult to create such a model. Information obtained from animal model experiments is used to solve health-related complications in humans and treat diseases. Therefore, perhaps it would be more appropriate to call laboratory animals “human models” [1].

**Figure 1:**
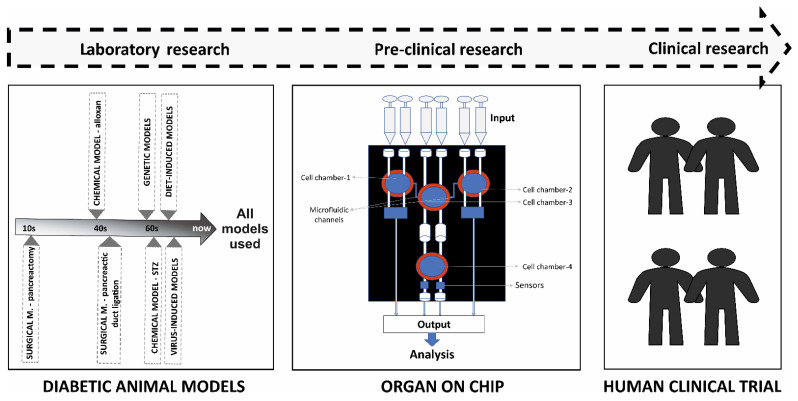
Pictorial diagram representing the concept of using organ-on-chip (OoC) in pre-clinical research and creating a bridge between drug discovery phase and human clinical trials.

In this review article, we have discussed the progress of diabetes research from the commonly used diabetic animal models and their contributions to scientific research to the recently developed concept of OoC (organ-on-chip) microdevices in diabetic research (Figure 1). The OoC technology, combined with iPSCs, has shown a huge potential to be used in research as a realistic disease model, as well as a potential to bridge the gap between pre-clinical research and human clinical trials [2].

**Figure 2:**
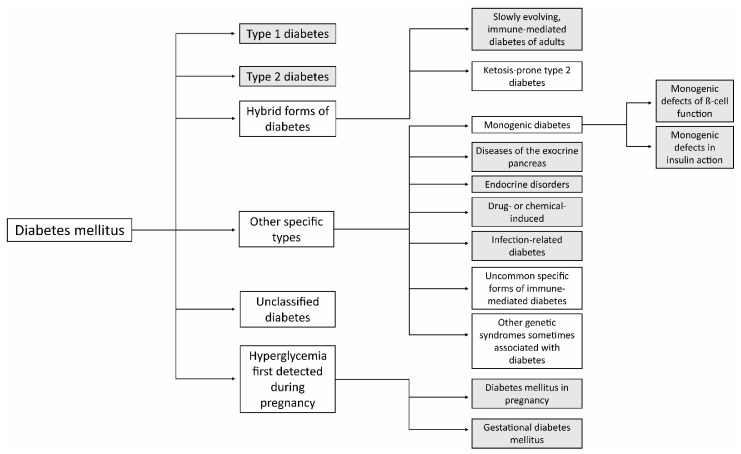
Classification of DM in humans processed according to classification of DM by World Health Organization, Geneva 2019. The highlighted types of DM can be modeled in laboratory animals.

Diabetes mellitus (DM) is a very serious disease of sugar metabolism, which can be caused by insufficient insulin production. That is referred to as Type 1 DM (T1DM). If it is caused by tissue resistance to insulin, it is referred to as Type 2 DM (T2DM). Specific types of diabetes include monogenic diabetes syndromes, diseases of the exocrine pancreas such as cystic fibrosis or pancreatitis, different endocrinopathies, and drug-induced diabetes. Another type of DM is called gestational diabetes (GDM), which is associated with high blood glucose during pregnancy [3]. The current classification of individual types of DM in humans, according to the World Health Organization (WHO), is shown in Figure 2.

DM is very widespread worldwide, affecting approximately 537-million adult people in 2021. The incidence of this disease in the adult population is rising rapidly; 541-million adults are now at an increased risk of developing T2DM. The International Diabetes Federation estimates that DM incidence will rise to 643 million by 2030 and predicts that DM will be the seventh-leading cause of death worldwide at that time [4,5].

## 2. History of Animal Models of Diabetes

Experimental animals were used for DM research as early as the second half of the 17th century, but the most important knowledge about the pathophysiology of this disease was only obtained at the turn of the 19th and 20th centuries. The significance of the pancreas in the pathophysiology of DM was discovered by a pancreatectomy that was performed on a dog by Josef von Mehring and Oskar Minkovski in 1889 [6]. This dog was the first described animal model of DM, although as early as 1683, Conrad Brunner described an experiment in which he removed the pancreas in a dog, which resulted in polyuria. In retrospect, it can be concluded that this polyuria was a symptom of the diabetic state [7]. In the second half of the 1970s, it was definitively recognized that there are two different types of diabetes with different pathophysiologies: T1DM, otherwise referred to as insulin-dependent DM, and T2DM, also called insulin-independent DM [8]. This was followed by the efforts of scientists to obtain an animal model corresponding to T2DM. Animal models reflecting human DM are of great importance in both basic and pre-clinical research. They help us not only to understand the mechanisms of this disease, but also to evaluate new therapeutic approaches. Unfortunately, creating an animal model reflecting human T2DM is very difficult. However, it is very important for comprehensive research on diabetes [9].

## 3. Surgical Models

An animal model was established through a series of experiments as only a complete pancreatotomy is a guarantee of the induction of significant DM in dogs. The exact procedure for pancreatectomy, in order to obtain a diabetic dog, was described in 1891 by Marcel Eugene Emile Gley [10]. Performing a subtotal pancreatectomy does not make it possible to predict the severity of the resulting metabolic disturbances in advance. It was found that just one-seventh of the pancreas is able to ensure the proper regulation of glucose metabolism in dogs [11]. Pancreatomy as a means of inducing diabetes in laboratory animals was used not only in dogs, but also in other animals such as cats, rats, mice, chicks, sheep, ducks, guinea pigs, and pigs [12,13,14,15,16]. Subtotal pancreatomy removing 95% of the gland in rats was described in 1944 by Foglia [17]. Later on, subtotal pancreatectomy was combined with the parenteral application of glucose in order to induce severe diabetes [18].

Over the following decades, a surgical model of diabetes, the pancreatotomy, was used in dogs. However, the trend of using diabetic animal models has changed from the 1940s when researchers started to use rodent models for diabetic research; these models have dominated until now. The dog surgical model is still in use, but only a few scientific articles have been published in the last few decades, as is evident from articles in the PubMed database. Recently, the pig model of diabetes induced by pancreatectomy has come to the fore, which allows for the study of gestational diabetes. It could also be used as one of the best possible animal models for experimental therapeutic procedures [19,20].

Another method of surgically inducing DM was described in rabbits as early as 1946 when Walpole and Innes performed a pancreatic duct ligation, which resulted in morphological changes of the islets and a slight rise in the fasting blood glucose level. Later, this model was investigated in more detail and assigned to T2DM models [21,22].

A surgical model was also used to obtain animals in a prediabetic and early diabetic state. In such a case, just 90% of pancreatic tissue has been excised [23].

## 4. Chemical Models

Inducing DM in an animal by administering a substance with a diabetogenic effect is an easy and relatively inexpensive way to obtain an experimental model of DM. This method began to be used in the first half of the last century and is still very popular in diabetic research. A wide range of substances exhibits a diabetogenic effect, including alloxan, streptozotocin, dithizone, potassium xanthate, monosodium glutamate, gold thioglucose, ferric nitrilotriacetate, uric acid, and lithium, of which only the first two are widely used [24].

In the mid-1940s, in addition to the previously used surgical model of DM, a chemical model induced by alloxan was also used. Alloxan was applied to rabbits [25], rats [26], monkeys, cats, and dogs [27]. Dunn and colleagues discovered that an alloxan application induces diabetes in animals by causing the necrosis of beta cells in the islets of Langerhans [28]. Twenty years later, another substance with a diabetogenic effect—streptozotocin (STZ)—was introduced [29]. Both alloxan and STZ are widely used diabetogenic chemicals in the research of diabetes [30]. They are predominantly used to induce T1DM in different animal species. They are both also glucose analogs with cytotoxic activity that are relatively unstable and must be administered parenterally [31]. The exact mechanism of the diabetogenic effect of these two substances was clarified gradually during the following decades after their introduction.

The selective uptake of alloxan by pancreatic islet β-cells was described in 1982 [32]. Later, it was discovered that alloxan, due to its steric similarity to the glucose molecule, can enter cells via the low affinity of the glucose transporter GLUT2 [33]. The subsequent necrosis of β-cells is caused by the generation of oxygen-free radicals [34]. STZ also uses the GLUT2 transporter to enter the cell, where it primarily causes DNA fragmentation and subsequent cell destruction [35].

These two substances were/are used to induce T1DM most often in mice and rats, as well as in rabbits, dogs, or pigs with comparatively less frequency [1]. However, their effect on pancreatic β-cells differs in some animal species. For example, in the guinea pig, STZ application causes permanent β-cell damage with a subsequent diabetic state, while alloxan administration induces symptoms of diabetes that disappear after two weeks due to β-cell regeneration [36]. T1DM can be induced in rats, mice, monkeys, pigs, and rabbits [31,37,38,39,40,41], whereas STZ, when administered to newborn rats, can induce T2DM [42].

The effect of alloxan and STZ administration was also studied in non-mammalian animals. In chickens, alloxan or STZ administration does not induce DM at any dose [43]. Castineiras with co-workers described that alloxan applied to frogs in non-lethal dose was not able to induce DM, while STZ application led to DM in half of the animals [44]. However, Kumar and Khanna demonstrated that the frog Rana tigrine is sensitive to alloxan [45], as well as to STZ [46]. Finally, STZ applied to another frog, Rana catesbeiana, did not cause islet degeneration [47]. The administration of SZT to Channa punctatus, a freshwater fish, induced some degenerative changes in β-cells but did not lead to DM [48], while alloxan application caused the necrosis of β-cells [49]. Pancreatic β-cells of tilapia, a teleost fish, are neither destroyed by STZ nor by alloxan [50,51]. In a zebrafish model, alloxan is predominantly used to induce DM [52], but the STZ model is also introduced [53].

The combined application of alloxan and STZ was used to induce DM in dogs. The advantage of this application is to use a lower amount of each of these substances and, thus, prevent damage to other organs [54,55]. Another option for safely inducing T1DM in a dog is a partial pancreatotomy with the application of a low dose of STZ [56]. In the same way, diabetes can be safely induced in a monkey [57]. Partial pancreatectomy, combined with the application of alloxan, could be used to induce DM in cats [58]. The induction of diabetes in Syrian hamsters can be administered by the application of alloxan, followed by a repeated administration of STZ. Alloxan alone does not cause chronic DM in this species [59].

The co-administration of STZ and nicotinamide to an adult mouse, along with a high-fat diet, induces T2DM in mice [60]. The co-administration of STZ and caffeine induces T2DM in young adult rats [61]. T2DM can also be induced in an adult rat by administering a low dose of STZ after feeding the rat with a high-fat diet [62,63]. Similarly, the same type of DM can also be induced in the mouse with comparatively lower doses of STZ administered repeatedly [64]. A slow infusion of high doses of STZ to pigs on a low-fat diet induces T2DM [65].

DM can also be induced in zebrafish by simply increasing the concentration of glucose in its environment without the application of other chemical compounds. The symptoms of diabetes persist even after the glucose is removed from the water [66]. The long-term administration of 30% D-galactose to young adult dogs or rats in their diet caused the development of retinopathy corresponding to morphological changes on the retina arising in connection with diabetes [67,68]. These animals can serve as a model of diabetic retinopathy, as well as diabetic neuropathy [69].

Since the 1950s, in addition to alloxan and STZ, dithizone, a zinc-chelating agent, has also been used to induce DM in cats, mice, rabbits, and golden hamsters [24,70]. Dithizone could cause the rupture of insulin-storing vesicles in pancreatic β-cells [71]. Currently, this substance is used to determine the purity of a sample of pancreatic islets before transplantation and/or to prove the presence of insulin cells [72].

Hyperglycemia and insulin resistance can also be caused by high levels of glucocorticoids. In mice and rats, this condition was induced by the application of dexamethasone [73,74]. However, the total number of studies using this model is very low compared to the frequency of use of other chemical models.

Over the decades, many modifications in protocols have been conducted and published for the chemical induction of diabetes in laboratory animals. These usually differ in the application dose or in the time schedule of the application. Some of the most commonly used chemical models of T1DM and T2DM are summarized in Table 1 and Table 2.

## 5. Virus-Induced Models

**Figure 3:**
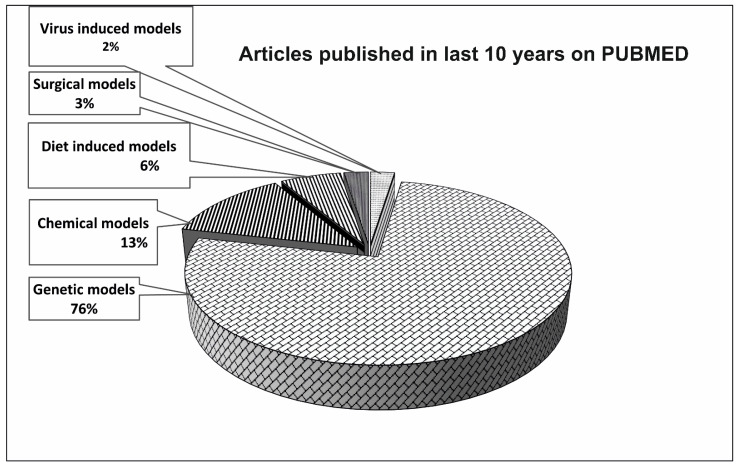
Understanding the importance of different types of animal models of diabetes through number of published articles on PUBMED in last decade. The reviews, systematic review, meta-analysis, and books/documents were excluded from the analysis. The search was done on 29 August 2023.

DM can also be induced in laboratory animals through a viral infection. The encephalomyocarditis (EMC) virus, reovirus Types 1 and 3, and the Coxsackie virus B4 were found to cause pancreatic islet necrosis in the mouse [87,88,89]. Mice of various strains, ages, and sexes were used to determine the effect of these viruses on pancreatic β-cells under different conditions. The effect of the congenital rubella infection was studied in rabbits [90]. It has been found that some viruses can damage β-cells and, therefore, cause diabetes, while other viruses prevent diabetes from occurring [91]. The induction of diabetes by a virus depends not only on the ability of a particular virus to induce diabetes, but also on the susceptibility of the host organism [92]. This is why it is not easy to create an animal model corresponding to virus-induced DM in humans. Neither the link between viral infection nor the development of T1DM has been clearly confirmed, nor has it been disproved. Therefore, it can be expected that a lot of attention will be paid to the creation of a suitable model. Within the last 10 years, only 2% of articles, which contain diabetic animal model research, include the virus-induced model (Figure 3).

## 6. Genetic Models

The essence of genetically induced DM models is a change in the genetic information of the experimental animal, which arose spontaneously or was purposefully created. In the 1960s, a spontaneous hereditary DM in Chinese hamsters was described [93]. Later, a non-obese (NOD) strain of mice spontaneously developing diabetes began to be used in the research of T1DM [94]. Spontaneous T2DM could also be developed in monkeys and dogs [95,96]. In the mid-1990s, animal models with a targeted mutation of a specific gene became available, which made it possible to study the importance of that specific gene in the pathogenesis of diabetes. These are most often transgenic and knock out mice [97].

Bio-breeding (BB) rats produce spontaneous autoimmune diabetes while also serving as NOD mice as one of the models for T1DM. They were derived from Wistar rats in 1974 [24]. The autoimmune destruction of β-cells is also typical for Long–Evans Tokushima lean rats (LETL). Its substrains, such as Komeda diabetes-prone rats and LEW.1AR1/Ztm-iddm are also raised from Lewis rats by a spontaneous mutation [98,99,100]. However, some animal models such as Chinese hamsters and Keeshond dogs [101], as well as New Zealand white rabbits [102], are less frequently used in Type 1 diabetic research.

Models of DM with naturally occurring mutations include non-obese diabetic mice, Lep^ob^ with mutation in leptin gene, and Lep^db^ mice with mutation in leptin receptor gene [103]. The mutation of the leptin receptor is also a characteristic of the frequently used rat model of diabetes, which is the model for Zucker diabetic fatty (ZDF) rats. Lep^ob^ and Lep^db^ mice, as well as ZDF rats, are used in T2DM research. The Goto–Katazaki (GK) rat is not obese, and it has a decreased mass of β-cells and insulin resistance [104]. Sand rats (*Psammomys obesus*), when fed a high-fat diet, will develop T2DM characterized by insulin resistance and a gradual loss of β-cells [105]. The Otsuka Long–Evans Tokushima Fatty (OLETF) rat strain was developed from Long–Evans rats’ strain and exerts T2DM, which is later (at about 40 weeks of age) replaced by T1DM [106]. The Cohen diabetic rat is a nonobese experimental model with many similarities with human pathophysiology of T2DM [107]. Spontaneous diabetic Torri (SDT) rats are the nonobese model of T2DM, which is caused by fibrosis of pancreatic islets followed by the destruction of β-cells with consequent insulin hyposecretion. Islet α-cells remain functional, contributing to the exacerbation of the diabetes [108]. A cross between a ZDF rat and a polygenetically obese Crl:CD rat produced rats designated Zucker diabetic Sprague-Dawley (ZDSD), which are obese and develop prediabetic metabolic syndrome, followed by T2DM with an intact leptin pathway [109]. Since the leptin pathway is intact in this model, it is a model that better represents the situation in the human organism, where damage to the leptin pathway in diabetics is also rare [110]. Another rat strain closely related to ZDSD rat exists and is designated as University of California Davis T2DM (UCD–T2DM) [111].

Polygenic mouse models of T2DM include several mouse strains. Kuo Kondo (KK) mice exert T2DM and obesity [112]. A congenic strain of KK mouse, KK/Ay, develops a more severe case of diabetes than original KK [113]. The Tsumara Suzuki obese diabetic mouse (TSOD) was established by Suzuki and co-workers in 1992 [114]. The New Zealand obese (NZO) mouse has already been used in diabetic research for more than 60 years [115].

The genetic selection also helps to develop two lines of Yucatan miniature swine with decreased (“low K”) or enhanced (“high K”) glucose tolerance. Impaired glucose tolerance was caused by decreased insulin secretion in response to glucose stimulation. Additional feeding of these pigs with high-fat diet causes higher glucose intolerance [116]. Another diabetic pig model, Göttingen minipig, was developed by crossbreeding the Vietnamese, German improved Landrace and Hormel swine in the 1960s [117].

Since the aforementioned animal models do not completely model the pathophysiological processes occurring in a diabetic patient, scientists are trying to create a more accurate model with the help of artificially induced mutations. A large number of such models are currently available. Most often, these are mouse models, as they are the easiest to prepare. In this article, we will mention the most frequently used ones.

By the end of the ‘90s, models with a genetic mutation of the gene necessary for insulin production were available, the most prominent representative of which are AKITA mice [118]. Akita mice, nonobese B6 mutant mice, spontaneously develop early age-onset diabetes, which is characterized by a low plasma level of insulin caused by a decrease in the number of β-cells without insulitis [118], therefore serving as a model of T1DM.

Mouse models transgenic for human islet amyloid polypeptide (IAPP) allow for the studying of the importance of this polypeptide in the pathogenesis of diabetes. Typically, in a patient with T2DM, this polypeptide is increased in the islet β-cells, but this is not the case in rodent models of T2DM [119]. Therefore, this transgenic model is very beneficial for studying the pathophysiological processes associated with diabetes in the human organism.

NSY mice have congenital diabetes developed in the descendants of STZ-induced diabetic mice. They exert glucose tolerance but do not have obese tendencies [120]. This model is reported to be more comparable to human T2DM in terms of disease progression compared to other mice models [121]. NSY mice were used to construct two novel congenic strains homozygous for different segments of NSY–Chr 14, a place where multiple genes for diabetes-related phenotypes are localized [122].

Recently, NSY mice have been used in the development of another mouse model of T2DM, which is characterized by early onset and persistent hyperglycemia, as well as obesity. This is caused by a spontaneous mutation of the agouti yellow gene. This strain is designated as NSY.B6-Tyr^+^,A^y^ [123].

## 7. Diet-Induced Models

The effect of a special diet on diabetic organisms was already described in 1947 by Houssay and Martinez [124]. Feeding a special diet is used to induce T2DM in some animal species. This was described in the 1960s in a sand rat (*Psammomys obesus*) that was fed commercial laboratory rat chow. These rats become obese, and they had signs of diabetes, including hyperglycemia, glycosuria, ketosis, and cataract, while other sand rats remain normal if fed with vegetables only. Subsequently, pancreatic β-cell degeneration was demonstrated in obese groups of animals [125]. A similar experiment was performed with albino rats, which were fed with a high-energy diet. After a few weeks, these subjects developed obesity and metabolic changes such as hyperinsulinemia and hypertriglyceridemia, but plasma glucose concentration remained normal [126]. Contrary, Sprague–Dawley rats fed a 30% fat diet for more than one week exerted hyperphagia, obesity, hyperinsulinemia, and hyperglycemia, which were positively corelating with fat intake [127].

A persistent high-sugar diet, as well as a high-fat/high-sucrose diet, can develop DM with limited glucose tolerance in rabbits [128,129]. In minipigs, high-fat/high-sucrose feeding induces insulin resistance, the dysfunction of β-cells, diabetes, and atherosclerosis [130]. Other studies showed that feeding high-fat/high-sucrose/high-cholesterol diet to Chinese Bama minipigs can also induce T2DM [131]. Chen and his colleagues demonstrated that pigs of different strains have different sensitivity to a special diet. Thus, the ability to induce diabetes is based on it. They have tested three strains of Chinese minipigs and determined that Wuzhishan and Bama minipigs are relatively susceptible to DM induced by high-fat/high-sucrose diet, while Nongda pigs are relatively resistant [132]. However, diet-induced DM in pigs is mild, approaching the human prediabetic condition or metabolic syndrome [133].

C57BL/6J mice, when fed a high-fat or high-fat/high-simple carbohydrate/low-fiber diet, produces moderate hyperglycemia and marked hyperinsulinemia, which is associated with hypertrophy and hyperplasia of islet tissue [134,135].

In spiny mice (*Acomys cahirinus*), feeding a standard laboratory rodent diet supplemented with high-oil seeds induces obesity and glucose intolerance accompanied by β-cell hyperplasia with intermittent hyperinsulinemia, which, after a few months, results in pancreatic islet collapse with subsequent insulin loss and ketosis [136].

Quite recently, it was determined that nile grass rats (*Arvicanthis niloticus*) spontaneously develop hyperglycemia and obesity within 1 year of their life when fed a conventional lab diet. This model is suitable to be used to study pathophysiological processes included in T2DM and metabolic syndromes [137].

The administration of a high-energy diet to laboratory animals in order to induce diabetes is also used in combination with the application of lower doses of STZ, which leads to the development of T2DM. This method of inducing T2DM in laboratory animals is more cost-effective than diet-only induction, as it significantly shortens the time required to induce diabetes [138].

As it follows from the previous information, there are many genetic models of DM of both types. By conducting a survey of the PubMed database, we found that in the last 10 years, some genetic models were used in three-quarters of all published diabetes research that used an animal model, while chemical models were used in 13% and others were used in less than 10% (Figure 3).

## 8. Gestational Diabetes Models

Changes in glucose metabolism induced by pancreatectomy in pregnant female dogs were already studied at the beginning of the last century. However, the results of the studies at that time were not clear-cut [139,140]. Information about the negative effect of pregnancy on the maternal organism’s glucose metabolism began to appear in the literature in the early 1970s. In 1979, the WHO described GDM as another type of diabetes [141]. Several animal models of GDM have been described so far, but none of them show a pathophysiology that is completely identical to humans [20]. Various modifications of the procedures outlined above can be used to induce GDM in animals. These include, for example, total pancreatectomy in late pregnancy, the application of alloxan or STZ during pregnancy, the administration of a high-carbohydrate and high-fat diet, or the use of genetic manipulation methods. A very detailed description of these methods can be found in the recently published high-quality review article [141].

## 9. Experiments on Animals in the 21st Century

In recent years, there has been increasing pressure for ethical reasons to reduce the number of animals used for research purposes. In the context of diabetes research, the use of rodents to model diabetes in humans has been found to be suboptimal due to a number of differences in the physiological processes related to glucose metabolism in rodents and humans [142]. Advanced technologies now allow us to simulate the in vivo environment much better than before, but we are still unable to simulate this environment perfectly. For this reason, it is still necessary to use animal models in research. However, it is still very important to find ways to reduce the number of laboratory animals. A consistent adherence to the 3R method helps with this. The following chapter describes already existing alternatives that have the potential to replace some experiments on animals. Their further improvement is the right way to reduce the need for laboratory animals in diabetes research.

## 10. Organ-on-Chip Models in Diabetic Research

Humans, when compared to animals, have different physiology, different types of organ functioning systems, different types of immune systems, and different cellular compositions of the organs and gene expression [143]. Moreover, it has been observed that variations in islet architecture between species may reflect the evolutionary acquired adaptation induced by altered physiological conditions. A concern has been raised regarding the interpretation of data based on rodent animal models to human studies because of a different islet architecture [144]. Due to this reason, conventional diabetic animal models may not be a sufficient model to provide relevant information. The number of drugs that fail during human clinical trials sometimes show toxicity or a lack of efficacy. However, similar drugs had very promising data in pre-clinical research on animal models. For example, many therapies are validated in multiple rodent models of painful peripheral diabetic neuropathy but failed to exhibit therapeutic efficacy during human clinical trials [145].

In the last two decades, the idea of Organ-on-Chip (OoC) microdevices has been successfully formulated due to new advancements in the field of microengineering [146]. The OoC platform has been created by using an integrative approach of different technological supports combining cell biology, engineering, and biomaterial technology along with the advances of microfluidics to recapitulate an environment to mimic the physiology or the pathophysiology of the specific organ. Moreover, the integration of biophysical devices to the traditional tissue culture system has improved the field. Newly designed devices may provide an environment where more than one cell can be grown, and cells within the extracellular matrix (ECM) cross-talk can be maintained. These integrations are very useful because they reduce the discrepancies between in vivo and in vitro environments.

In 2004, Sin and co-authors demonstrated, for the first time, a detailed description of multi-OoC systems [147]. They developed the concept of “organ-on-chips (OoCs).” In 2010, Huh and co-authors developed “lung-on-a-chip” by reconstituting organ-level lung functions on a chip [148]. Numbers of OoCs are created, which are lined up with living cells and mimic the microarchitectures of specific tissues and organs. These include pathological models for gut-on-a-chip [149] liver-on-a-chip model [150,151], kidney-on-a-chip [152,153,154,155] and lung-on-a-chip [156]. Likewise, new developments in the field of biomedical engineering have made it possible to create a microfluidic-based platform for in vitro drug screening. In the field of diabetes, a number of unique in vitro models have been developed, which can recreate native islets and can be used to study the pathophysiology of diabetes or the understanding of the pharmacology of new drugs.

**Figure 4:**
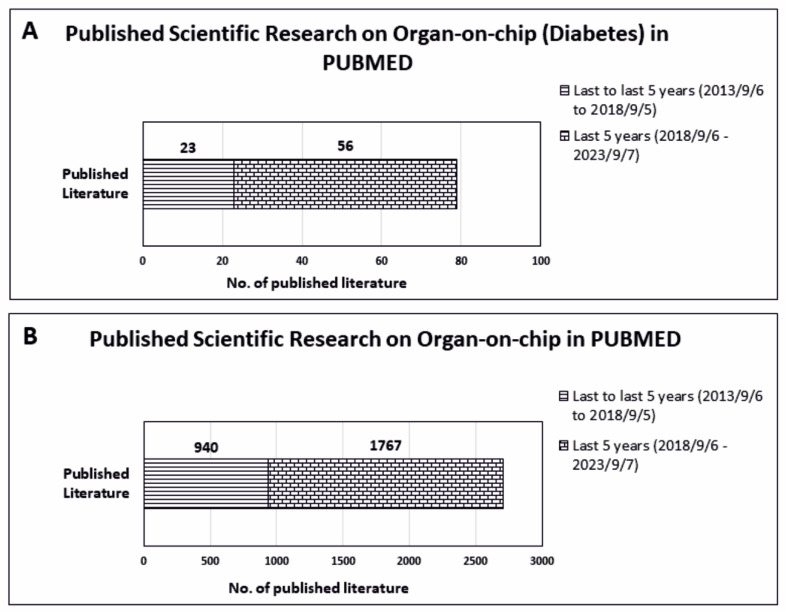
Understanding the importance of Organ-on-Chip (OoC) in last decade: Data analysis was done on PUBMED (**A**) by using keyword “Organ-on-Chip” for last 5 years (6 September 2018–7 September 2023, ) versus last 5 years (5 September 2013–6 September 2018), and (**B**) by using keyword “Organ-on-Chip,” along with sub-filter “Diabetes” for last 5 years (to 6 September 2018–7 September 2023) versus last 5 years (to 5 September 2013–6 September 2018). The reviews, systematic review, meta-analysis, and books/documents were excluded from the analysis. The search was conducted on 18 September 2023.

To understand the scientific contribution in the field of OoCs devices, we have investigated the number of published articles on PUBMED MEDLINE database using the specific key word “Organ-on-Chip” for the last 5 years, 10 years, and total published literature. For analysis, we have excluded the reviews, systematic review, meta-analysis, and books/documents so that the real scientific contribution of authors can be estimated. We have observed a drastic increase in publications in the last 5 years (6 September 2018–7 September 2023). It is ~1.9 times more if compared to publications of the last 5 years (5 September 2013–6 September 2018) (Figure 4B). In addition, we have extended our search by applying another sub filter, “Diabetes.” Interestingly, we have found only 56 published articles in the last 5 years (6 September 2018–7 September 2023), which is ~2.4 times more than publications published in the last 5 years (5 September 2013–6 September 2018) (Figure 4A).

## 11. Insulin Resistant Adipose Model

In 2005, Nguyen and co-authors demonstrated that fat-induced insulin resistance can be developed in 3T3L1 cells by treating the cells with a mixture of free fatty acids (FFAs). It has been observed that FFA treatment can impair insulin receptor-mediated signal transduction, which may alter GLUT4 translocation and can diminish glucose transport and finally induce cellular insulin resistance via the activated JNK pathway or via TNF-alpha-independent mechanisms [157]. On the contrary, Ruan and co-authors have developed a model of insulin resistant in adipocytes by using TNF-α on differentiated 3T3–L1 cells in 2002 [158]. In 2003, Rotter and co-authors developed insulin-resistant models by inducing IL-6 into differentiated 3T3–L1 cells [159]. Many efforts have been made by a number of authors to develop improvised models of insulin resistance [160,161]. In 2019, Liu and co-authors have used a dynamic microphysiological in vitro model of the human adipose tissue to study the adipocyte–immune cell interaction in T2D [162]. This dynamic OoC is created in such a way so that it mimics the microvascular network of in vivo condition. Under this condition, the nutrient can be supplied and maintained in a constant rate along with waste removal. Human pre-adipocytes cells can be seeded on the chip, which are fully differentiated into adipocytes and maintained for several weeks. Upon the co-culturing of U937 cells, adipocytes developed a tendency for insulin resistance. Moreover, the author has demonstrated that the microfluidic system has the potential to study the obesity associated with insulin resistance and T2D. Subsequently, Kongsuphol and co-authors have developed an in vitro microfluidic-based model for immune-metabolic analysis to understand the micro-physiology processes of the inflamed human adipose tissue in obesity-associated T2D. The perfusion-based microfluidic device is designed in such a way that different types of cells, adipocytes/PBMCs, could be co-cultured, and their communication could be studied. The inflammatory cytokine profile is studied to understand the mechanisms of inflammations. Moreover, the effect of different types of diabetic drugs such as metformin, neutraceutical compounds, and omega-3 have shown comparable results to previously reported data [163]. Recently, in 2020, Tanataweethum and co-authors established an in vitro adipose-chip model, which displays superior cell function over conventional cell culture. Insulin resistance (IR) was induced into primary adipocytes by using TNF alpha. Cells were further maintained, differentiated, and induced in a microfluidic OoC. Moreover, the author has also validated the impact of the diabetic drug “rosiglitazone” on insulin-resistant adipocyte cells [164]. In conclusion, this model exhibits key characteristics of IR and can be used as a platform for discovering new drugs and understanding the signaling transduction of insulin resistance in T2D. The above-mentioned OoC device can be used for screening and testing new diabetic drugs for T2D.

## 12. Glomerulus-on-a-Chip

Many investigators have reported that a microfluidic kidney-on-a-chip, which could be developed by fabricating a sandwich chip lined with one or two types of renal tubule cell lines, can be used for toxicological research [165,166,167]. In 2010, Jang and Suh have developed a multi-layer microfluidic device to culture and analyze the renal tubular cells. Rat inner medullary collecting duct (IMCD) cells are cultured inside the channel, which generates an in vivo-like tubular environment for the cells [153]. In 2011, Jang and co-authors have studied the role of fluidic environment such as fluidic shear stress, hormonal stimulation, and the transepithelial osmotic gradient, which regulates the reorganization of intracellular F-actin using a microfluidic device [166]. Moreover, different authors have contributed in many ways to prepare a suitable in vitro/in vivo organ-level disease model “glomerulus-on-a-chip,” which is capable of mimicking and regulating the complex mechanical processes [168]. In 2017, Wang and co-authors developed a microfluidic device that reconstitutes organ-level kidney functions and mimics the early stages of diabetic nephropathy in humas-on-chip [169]. The OoC microdevice was able to exhibit critical pathological responses of diabetic nephropathy in glomerulus in vitro, which were similar to high-glucose-induced diabetic nephropathy in humans, such as GFB dysfunction, ROS production, and protein leakage responses. Therefore, it provides a unique platform for studying the mechanism of diabetic nephropathy and developing an effective therapy in glomerular diseases.

In 2019, Petrosyan and co-authors have established a model of Glomerulus-on-a-Chip (GOAC) by using human podocytes and human glomerular endothelial cells (hGEC) seeded on Organoplates^TM^ (MIMETAS) [170]. The device was developed to handle cell culture for a longer time with persistent phenotypes. Interestingly, glomerular cells on a chip can also interact and produce layers of extracellular matrix composed of Collagen IV and laminin, the main constituent of the glomerular filtration barrier in vivo. To validate the functionality of GOAC, the author has used serum from individuals affected by different glomerular diseases, including membranous nephropathy (MN) and evaluated the drug response. In 2020, Xie and co-authors have developed an extruded topographic hollow fiber (h-FIBER) in microfluidic devices, which resembles a vessel-like perfusable tubular channel for the cultivation of endothelial cells and a glomerulus-like knot with micro convex topography on its surface for podocyte cultivation [171].

Recently, Perin and co-authors have developed a device “Glomerulus-on-a-Chip” system that mimics phenotypically and functionally the in vivo glomerular microenvironment, which also responds to injury. The device has shown the ability for free diffusion of insulin and impermeability to albumin in physiological concentrations. The study has confirmed that exposure to nephrotoxic agents, such as puromycin aminonucleoside or sera from patients with anti-podocyte autoantibodies, can lead to albumin leakage similar to in vivo conditions. It also confirms that the device can replicate in vivo glomerular microenvironment with both phenotypical and functional manners [172].

## 13. Pancreas-on-Chip

Human Pancreas-on-a-Chip (PoC) could be considered as a quick evolving platform islet physiology that has the potential for clinical islet transplantation. Moreover, a number of PoCs have been developed to study the microenvironment of the pancreas, as well as islet functions. In 2016, Xing and co-authors developed a microfluidic platform and used pancreatic islets from both rodents and humans [173]. In 2017, Bauer and co-authors developed a microfluidic two-organ-chip model for the co-culture of human pancreatic islet microtissues and liver spheroids [174]. This ex vivo T2D model is useful to study the pancreatic islet–liver cross-talk and glucose regulation.

In 2020, Rodriguez–Moncayo and co-authors designed a microfluidic-based platform to estimate cytokine secretion such as interleukin-8 and tumor necrosis factor-alpha secretion from a consistent number of blood-derived monocytes, neutrophils, and THP-1 monocytes. The device was able to handle and analyze both types of cultures adherent and nonadherent in 32 cell culture chambers, and each pattern has 492 microwells [175]. In 2022, Rodríguez–Comas and co-authors summarized the most relevant studies related to pancreatic islets and microfluidics, focusing on the molecular and cellular-scale activities that underlie pancreatic β-cell function. A variety of microfluidic devices have been presented to recreate the microenvironment of the pancreas to study islet function. Different functional assays provided further information regarding the use of microfluidics as an excellent tool to perform comprehensive islet analysis and to obtain a more significant predictive value for islet functionality [176]. Interestingly, Abadpour and co-authors have reviewed and summarized the new advancements and challenges in the field of OoC devices related to PoC. The use of PoC in drug development, islet research, and clinical islet transplantation is also discussed [177].

## 14. Diabetic Foot Ulcer on Chip

Revolution in the field of bioengineering has tremendously changed the platform of traditional cell culture systems to OoC where technology has been permitted to design in vitro models for skin. The reconstruction of in vitro models of skin has a weak skin barrier function compared to normal human skin. It is due to a compromised system for mechanical forces and dynamic flow, which can ultimately impact the mechanistic signals, supply, and drainage of nutrients and metabolites. In 2018, Sriram and co-authors designed and reported a full thickness human Skin-on-Chip (SoC) microfluidic model with a dynamic flow and epithelial tissue culturing system. The designed SoC device has shown a better recapitulation of skin if compared to conventional static culture systems. The device can also integrate with 3D culturing, along with the ability to test the integrity/permeability directly on the device with higher throughput and the automation of culture [178]. In 2019, Lukács and co-authors designed a Skin-on-a-Chip microfluidic device and compared the absorption of caffeine via penetration assays [179]. In 2021, Ejiugwo and co-authors developed an immunocompetent diabetic foot ulcer (DFU) model onto a microfluidic platform. The DFU model has pathophysiological conditions, which can recapitulate the contribution of macrophages, fibroblasts, and keratinocytes in wound healing [180]. This model may be considered as an ideal and robust in vitro platform for wound-healing therapeutics.

## 15. iPSCs and Organ-on-Chip

The integration of stem cell research with microfluidics has provided a unique platform to create a disease model on a chip-based microdevice. This new avenue of scientific research has revolutionized the drug development pipeline, where patient-derived iPSCs can be grown on 3D matrices by using hydrogels, micromachining, and advanced 3D printing inside the microfluidic devices. The OoC technology, combined with iPSCs, shows great potential to create more realistic disease models, which can be used as a bridge between pre-clinical research and human clinical trials [2]. In addition, these models can be used for the discovery of new drug molecules and screening of best candidates for human clinical trial. In 2022, Fanizza and co-authors have reviewed and elaborated the role of iPSCs in the screening of drugs for personalized medicine, especially on neurodegenerative diseases. The translational value of OoC was also analyzed to create more realistic disease models [181].

The pharmacodynamic and pharmacokinetics (PK–PD) of the drug can be studied in similar physiological conditions by using single-OoC or multi-OoC or Body-on-a-Chip. Moreover, the multi-organ cross-talks can also be facilitated in multi-OoCs to create similar physiological conditions and organ functions, as in humans [182,183]. These multi-OoC models can be used to analyze the efficacy and safety of the drugs on an individual basis and may lay the foundation for developing next-generation OoCs with the concept of developing “Patient-on-a-Chip” [184].

Sung and co-authors have developed a novel approach by combining in vitro experiments to mathematical modeling approach for PK–PD analysis and developed a “PK–PD Model-on-a-Chip.” Authors have claimed that a combined in vitro/in silico approach could predict drug toxicity in a more realistic manner rather than a conventional way. The potential impact of any drug and its toxicities such as ADME (absorption, distribution, metabolism, and excretion) can also be analyzed using multi-OoC models [185].

In 2017, Musah and co-authors demonstrated that microfluidic OoC could be developed by using h–iPSCs into podocytes, which showed >90% efficiency and also express specific markers such as nephrin, WT1, and podocin. This in vitro model has an ability to recapitulate in vivo physiology of kidney glomerular and disease states such as adriamycin-induced albuminuria and podocyte injuries. Therefore, this device may facilitate and help in drug development and personalized medicine [186]. Likewise, the patient-specific organoid tubule “OrganoPlate^®^ microfluidic device” is created by using iPSC derivations [187]. Similarly, iPSC-derived cardiomyocytes have been used to develop Heart-on-a-Chip [188,189,190]. The most common cause of drug failure is drug-induced hepatotoxicity. Recently, iPSC-derived liver organoids are used to study drug metabolism, detoxification, and hepatotoxicity on chip. In 2018, Wang and co-authors coupled OoC with iPSC-derived liver organoid technologies and developed a 3D perfusable chip system to simplify the organoid production and recapitulating the condition of human liver in situ [191]. In 2021, Sakolish and co-authors developed a four-cell liver acinus microphysiology system (LAMPS) and four cell lines such as endothelial, Kupffer, and stellate cells and primary human hepatocytes, or iPSC-derived hepatocytes. A number of drugs, including Rosiglitazone, Pioglitazone, and Troglitazone, were tested. They observed that both primary hepatocytes or iPSC-hepatocytes in 3D culture have an excellent basal liver function [192].

Recently, a combined effort has been made by the National Institute of Diabetes and Digestive and Kidney Diseases and the National Center for Advancing Translational Sciences to support research related to human tissues on chips. They have funded numbers of projects to support the idea for developing an integrated in vitro model of immune-mediated metabolic dysfunction for human T2DM [193].

The value of OoC has been tremendously enhanced by using patient-specific iPSCs for developing a new generation device called “Patient-on-a-Chip.” These types of devices can be used to study the cross-talk between varieties of cells of different origins because they mimic a disease condition similar to in vivo conditions in humans. This unique platform can also be developed as a platform for screening newly developed drugs prior to human clinical trials.

## 16. Conclusions

Preclinical experimental models in diabetic research are a fundamental tool to conduct basic research and improve our knowledge of the disease. The role of these models in diabetic research is enormous and cannot be adjudicated. For example, in early 1980, two analogues of clofibrates such as thiazolidinedione and ciglitazone were developed by Takeda Pharmaceuticals in Japan. The analogues were tested in rodents and caused edema and lenticular opacities. In 1997, troglitazone was the first thiazolidinedione to be approved for clinical use. However, after some time, it was found to cause liver damage and was withdrawn from the market by the US Food and Drug Administration in 2000. However, ciglitazone was never marketed because it showed a mild effect on glucose production [194]. It is well-known that every animal model has its own limitations. The most indispensable difference is their physiology, which is different from human. Moreover, it has been observed that no experimental animal model, such as surgical models, chemical models, virus-induced models, genetic models, diet-induced models, etc., can completely resemble human diabetic conditions. Due to this reason, conventional diabetic animal models are not able to provide whole information regarding the pathophysiology of the disease, since the pathophysiology and molecular mechanisms of DM are not fully explained in humans. Therefore, more studies are required to develop new diabetic models to facilitate and develop a platform for a better understanding of the disease. Recently, an integration of biophysical devices to the traditional tissue culture system has been used, which has transformed the conventional way of cell cultures to more advanced ways by using microfluidics chips, where human cells can be grown on a chip and used for in vitro drug analysis.

The idea of OoC has been developed and established in the last two decades. An integrative approach has been used, which combines interdisciplinary approach to develop microfluidic devices for cell culture. The OoC microdevices are developed and used to recapitulate an environment to mimic the physiology or pathophysiology of the specific human organ. Moreover, designed OoC devices may provide an environment to grow more than one type of cell type and also facilitate different types of cells to cross-talk. Interestingly, a unique opportunity is being developed by using stem cell research on microfluidic devices, where induced human cells are grown in OoCs to understand the pathophysiology of the disease and drugs screening.

In conclusion, new advancement in the field of microfluidics may create some unique platform in future, which may bridge the gap between animal models used in preclinical research to human clinical trials. However, it may generate a new era of responsibilities and issues such as ethical–social issues, copyright-related issues, patent-related issues, etc. Consequently, new laws and standards must be approved for the usage of iPSCs on OoC microdevices because private information of the patient is present in the form of DNA, which should be protected. Moreover, the ethical integrity of iPSC production should also be ensured prior to its usage in the field of research. At least, the donor must be informed about the usage of their cells and consent must be obtained from him prior to the usage of their cells.

## Figures and Tables

**Table 1:** List of chemically induced DM Type 1 models in different animal species. Abbreviations: STZ streptozotocin; i.p. intraperitoneally; s.c. subcutaneously; i.v. intravenously.

Chemical	Animal	Dose	Reference
STZ	Mouse	Multiple low-dose: 40 mg/kg i.p. for 5 consecutive days	[75]
		Single high-dose: 100–200 mg/kg i.v. or i.p.	[76]
	Rat	Multiple low-dose: 20 mg/kg i.p. for 5 consecutive days	[77]
		Single high-dose: 40–70 mg/kg i.v. or i.p.	[75]
	Hamster	50 mg/kg i.p.	[78]
	Dog	25 mg/kg i.v.	[79]
	Pig	150 mg/kg i.v.	[80]
	Primates	50–150 mg/kg i.v.	[24]
Alloxan	Mouse	150 mg/kg i.p.	[81]
	Rat	125 mg/kg s.c.	[82]
		200 mg/kg i.p.	[83]
	Rabbit	100 mg/kg i.p.	[84]
	Dog	50–75 mg/kg i.v.	[24]
	Pig	100–175 mg/kg i.v.	[85]

**Table 2:** List of chemically induced DM Type 2 models in different animal species. Abbreviations: NA nicotinamide; STZ streptozotocin; i.p. intraperitoneally; i.v. intravenously; HFD high fat diet; FDS10 10% fructose drinking solution.

Animal	Chemical	Diet	Reference
Mouse	NA 240 mg/kg i.p. (15 min before STZ) STZ 100 mg/kg i.p. (twice on day 0 and 2)	HFD	[60]
Rat	NA 230 mg/kg i.p. (15 min before STZ) STZ 65 mg/kg i.v.	Normal	[24]
	STZ 35 mg/kg i.p. (after 2 weeks of HFD)	HFD (58% calories as fat)	[86]
	STZ 40 mg/kg i.p. (after 2 weeks of FDS10, then normal water)	FDS10	[63]
	Caffeine 20 mg/kg i.p. (15 min before STZ) STZ 65 mg/kg i.p.	Normal	[61]
Pig	STZ 130 mg/kg as 30 min slow infusion	Low fat diet (5% calories as fat)	[65]
